# Genetic diversity and genotype multiplicity of *Plasmodium falciparum* infection in patients with uncomplicated malaria in Chewaka district, Ethiopia

**DOI:** 10.1186/s12936-020-03278-6

**Published:** 2020-06-08

**Authors:** Abdulhakim Abamecha, Hassan El-Abid, Daniel Yilma, Wondimagegn Addisu, Achim Ibenthal, Abebe Genetu Bayih, Harald Noedl, Delenasaw Yewhalaw, Mohieddine Moumni, Alemseged Abdissa

**Affiliations:** 1 School of Medical Laboratory Science, Faculty of Health Sciences, Institute of Health, Jimma University, Jimma, Ethiopia; 2 Laboratory of Cellular Genomics and Molecular Techniques for Investigation, Faculty of Sciences, Moulay Ismail University, Meknès, Morocco; 3 Department of Internal Medicine, Institute of Health, Jimma University, Jimma, Ethiopia; 4 Faculty of Science and Art, HAWK University, Gottingen, Germany; 5 Armauer Hansen Research Institute, Addis Ababa, Ethiopia; 6 Malaria Research Initiative Bandarban (MARIB), Vienna, Austria; 7 Department of Biomedical, College of Public Health and Medical Science, Mettu University, Mettu, Ethiopia; 8 Tropical and Infectious Diseases Research Center (TIDRC), Jimma University, Jimma, Ethiopia

**Keywords:** Genetic diversity, Multiplicity of infection (MOI), *Plasmodium falciparum*, Ethiopia

## Abstract

**Background:**

Genetic diversity in *Plasmodium falciparum* poses a major threat to malaria control and elimination interventions. Characterization of the genetic diversity of *P. falciparum* strains can be used to assess intensity of parasite transmission and identify potential deficiencies in malaria control programmes, which provides vital information to evaluating malaria elimination efforts. This study investigated the *P. falciparum* genetic diversity and genotype multiplicity of infection in parasite isolates from cases with uncomplicated *P. falciparum* malaria in Southwest Ethiopia.

**Methods:**

A total of 80 *P. falciparum* microscopy and qPCR positive blood samples were collected from study participants aged 6 months to 60 years, who visited the health facilities during study evaluating the efficacy of artemether-lumefantrine from September–December, 2017. Polymorphic regions of the *msp*-*1* and *msp*-*2* were genotyped by nested polymerase chain reactions (nPCR) followed by gel electrophoresis for fragment analysis.

**Results:**

Of 80 qPCR-positive samples analysed for polymorphisms on *msp*-*1* and *msp*-*2* genes, the efficiency of *msp*-*1* and *msp*-*2* gene amplification reactions with family-specific primers were 95% and 98.8%, respectively. Allelic variation of 90% (72/80) for *msp*-*1* and 86.2% (69/80) for *msp*-*2* were observed. K1 was the predominant *msp*-*1* allelic family detected in 20.8% (15/72) of the samples followed by MAD20 and RO33. Within *msp*-*2*, allelic family FC27 showed a higher frequency (26.1%) compared to IC/3D7 (15.9%). Ten different alleles were observed in *msp*-*1* with 6 alleles for K1, 3 alleles for MAD20 and 1 allele for RO33. In *msp*-*2*, 19 individual alleles were detected with 10 alleles for FC27 and 9 alleles for 3D7. Eighty percent (80%) of isolates had multiple genotypes and the overall mean multiplicity of infection was 3.2 (95% CI 2.87–3.46). The heterozygosity indices were 0.43 and 0.85 for *msp*-*1* and *msp*-*2*, respectively. There was no significant association between multiplicity of infection and age or parasite density.

**Conclusions:**

The study revealed high levels of genetic diversity and mixed-strain infections of *P. falciparum* populations in Chewaka district, Ethiopia, suggesting that both endemicity level and malaria transmission remain high and that strengthened control efforts are needed in Ethiopia.

## Background

The intensification of malaria control interventions has resulted in its global decline, but it remains a significant public health burden across several malaria-endemic countries [1]. The 2018 global malaria report revealed that the incidence rate of malaria declined by 18% from 2010 to 2017, in the same period, the estimated number of cases dropped from 239 million to 219 million, and the number of deaths from 607,000 to 435,000 [1–3]. In Ethiopia, the trends in malaria over the past five years have also shown a decline in malaria cases and fewer epidemics [4, 6]. In 2014/2015, Ethiopia reported 2174,707 malaria cases and 662 reported malaria deaths among all age groups which is a 98% reduction compared to 41,000 estimated deaths in 2006 [4, 6]. Between June 2016 and July 2017, the Ethiopian Health Management Information System (HMIS) reported a total of 1,755,748 malaria cases and 356 deaths due to malaria [4]. The key interventions which have been contributing to such significant decline includes: introduction of prompt and effective treatment with artemisinin-based combination therapy (ACT), the distribution and promotion of the use of long-lasting insecticidal nets (LLINs), nationwide coverage of indoor residual spraying (IRS), and environmental management [4–6]. Ethiopia adopted artemether-lumefantrine (AL) in 2004 as first-line for the treatment of uncomplicated falciparum malaria, LLINs coverage has been scaled up in Ethiopia since 2005, resulting in over 64 million nets distributed by 2014. IRS, including permethrin, bendiocarb propoxur and deltamethrin, pirimiphos-methyl has been used between 2014–2020. Although these control measures have resulted in a substantial decrease in malaria infections in Ethiopia, malaria is still endemic, with populations in some areas remaining at high risk of infection. Ethiopia has set a goal to eliminate the disease by 2030 using these interventions [4, 7].

Genetically-distinct malaria parasites in natural populations have an extremely high rate of genetic recombination during the sexual stages in a mosquito host, often resulting in multiple strains being transmitted simultaneously [8]. This diversity hampers development of effective vaccine as it limits the efficacy of protective immunity (i.e., antibody–mediated parasite inhibition) [9]. Highly endemic malaria settings are prone to infections containing multiple *P. falciparum* strains, primarily due to repeated exposure to mosquitoes infected with multiple parasite strains [10]. This genetic diversity of the parasite is one of the main factors responsible for the slow acquisition (several years) of immunity against malaria. Thus, individuals would have to encounter a broad range of circulating parasite populations before they develop an effective anti-malarial immunity [11].

Genetic diversity and multiplicity of *P. falciparum* infections are essential parasite indices that could determine the potential impact on the selection of drug-resistant parasites. Although many polymorphic antigens have been described in several stages of the parasite life cycle, merozoite surface protein 1 and 2 (*msp*-*1* and *msp*-*2*) seem to be the most appropriate to distinguish parasite populations [12–14]. This markers are particularly useful in determining the multiplicity of infection (MOI), a measure of the effectiveness of intervention programmes and also *msp*-*1* and *msp*-*2* typing are widely used in anti-malarial drug efficacy trials to distinguishing recrudescent parasites from new infections [15–17]. Study reports by Jelinek et al. [18] and Meyer et al. [19] showed that increased genetic diversity of circulating malaria parasites in a population in-creases the potential for the selection of drug resistance.

Declining malaria transmission as a result of scaling-up interventions has been shown to affect the parasite population genetics pattern and population structure of *P. falciparum* [20–22]. The scale-up interventions, such as the usage of insecticide-treated bed nets, indoor residual spraying [21, 23] and the introduction of new anti-malarial drug regimens [20, 24–29] to control and treat malaria have been shown to cause the genetic drift and decrease the level of allelic diversity(*He*) and MOI. However, this does not occurred in all settings [20, 21]. In addition, the genetic diversity and population structure studies can be used to monitor the effects of any malaria scale-up interventions, such as the impact of malaria control and elimination programs [30**]**. Hence, accurate assessment of the parasite’s genetic diversity across malaria endemic regions could help plan or develop new control and elimination strategies. The MOI, which identifies the number of clones within a particular infection, can serve as a measure of the level of malaria transmission as well as identify hotspots [31, 32]. Malaria parasite diversity is distinct in different individuals, populations, transmission settings and seasons within endemic zones and changes with variations in parasite prevalence [33], and has been suggested to be constantly changing [34–37]. Parasite populations even respond to specific interventions, such as rapid diagnostic tests, human host immune pressure and mosquito vector [38–40]. The identification of hotspots is important in understanding the epidemiology of *P. falciparum* infections for informed interventions to be implemented [32, 41]. The effect of malaria control interventions on the *P. falciparum* population structure in Ethiopia could not be assessed due to the lack of genetic data and systematic genetic surveillance study. Chewaka district in Southwest Ethiopia experiences frequent epidemic outbreaks of malaria. Parasite genetic diversity and multiplicity of infection studies have also been found to be important in the surveillance of strains circulating in a particular transmission area especially in Southwest Ethiopia because there was so limited information available on the genetic structures of *P. falciparum* [42–44]. This study was aimed at characterizing the genetic diversity and allele frequencies of *msp*-*1* and *msp*-*2* genes of *P. falciparum* isolates from uncomplicated malaria patients in Chewaka district, Southwest Ethiopia.

## Methods

### Study setting

The study was conducted in Ilu-Harar Health Centre, Chewka district, Buno Bedele Zone, Southwest Ethiopia during September–December 2017. Chewaka district is located in Buno Bedele zone, Oromia regional state, Ethiopia about 570 kilometres southwest of Addis Ababa. It is situated in lowland areas of Dhidhesa valley, which lies below 1500 m above sea level. The district has 26 administrative *kebeles* (villages). As in most other areas, malaria transmission in Chewaka follows rainy seasons, with transmission peaking in the months between September and December and between April and May. The main malaria control strategy in the district includes long-lasting insecticidal nets (LLINs), indoor residual spraying (IRS) and malaria case management with ACT [3, 6]. In 2017, the FMOH updated the country’s malaria risk strata based on malaria annual parasite incidence (API), calculated from micro-plan data from more than 800 districts, classifying areas with malaria transmission risk by API as high (≥ 100 cases/1000 population/year), moderate (≥ 5 and < 100), low (> 0 and < 5), and malaria-free (~ 0). Chewaka district was classified as mesoendomic/moderate transmission setting [4].

### Study population and blood sample collection

A total of 80 *P. falciparum* infected blood spots were collected during a therapeutic efficacy study of artemether-lumefantrine (Coartem^®^), between September and December 2017. The PCR analysis of *msp1* and *msp2 gene* markers showed that three cases were recrudescence and a single case of re-infections was observed in the study. The observed recrudescent parasitaemia was between 14–21 days after treatment start. However, the recrudescent parasitaemia resolved quickly after initiated re-treatment in all cases with the same regimen (unpublished data). The participants were aged between 6 months and 60 years, were residents within Chewaka area, and had presented to the local health centre. Febrile patients with axillary temperatures ≥ 37.5 °C, positive for asexual *P. falciparum* mono-infection giving written consent were included in the study. Children aged less than 6 month, pregnant women and individuals suffering from any other diseases were excluded.

After consent was obtained, the blood samples were obtained by finger prick and malaria infection was diagnosed using microscopy and qPCR. Whenever a participant tested positive for asexual *P. falciparum* mono-infection, approximately 50 μl of whole blood was spotted onto filter paper (Whatman^®^ 927 mm) and air dried. The blood spots were individually placed into plastic bags with desiccant and transported to the Jimma University Clinical Trial Unit (JU-CTU) and stored at −20 °C for a maximum of 3 months prior to further analysis.

### Extraction of parasite DNA

Genomic DNA was extracted from whole blood using proteinase K-base method (GE Healthcare Illustra Blood Genomic Prep Mini Spin Kit) according to the manufacturer’s instructions for qPCR species identification and parasite density determination. For nested PCR, the DNA was extracted from stored dried blood spots collected on enrollment (Day-0) and on any day after day 3 were deemed to have recurrent parasitaemia using Pure Link^TM^ Genomic DNA mini Kit (Invitrogen, USA) according to the manufacturer’s instructions. DNA was checked for purity and quantity using Nanodrop spectrophotometer (ND 1000), and stored at −20 °C until used for PCR amplification and detection.

### Quantitative PCR (qPCR) screening for *Plasmodium falciparum*

Primer design genesig (Bio-Rad Laboratories, Inc. Germany) standard Kit for *Plasmodium* spp. genomes was analysed for the in vitro quantification of all *Plasmodium* spp. genomes by targeting the 18S ribosomal RNA (18S) gene according to the protocol of Primer design^TM^ Ltd [45]. Species specific *Plasmodium falciparum* genome was analysed for in vitro quantification of *P. falciparum* genomes by targeting the *plasmepsin 4* gene according to the protocol of Primerdesign^TM^ Ltd [46]. Each reaction was performed in duplicate and the cycle threshold number (Ct) was determined as their mean. A sample was considered positive if the fluorescent signal was detected in at least one replicate; conversely, if no signal was detected within 40 cycles, a reaction was considered negative.

### Genotyping of *Plasmodium falciparum* isolates

Genotyping of *P. falciparum* isolates was carried out by Nested PCR amplification of the two highly polymorphic regions of *msp*-*1* (block2) and *msp*-*2* (block3) genes as reported previously [47, 48]. Primer sequences (Additional file 1: Table S1 and Additional file 2: Table S2) and cycling parameters used for amplification of the three allelic families of *msp*-*1*(K1, MAD20 and RO33) and two allelic families of *msp*-*2* (FC27 and 3D7) have been reported elsewhere [49, 50]. Briefly, in the initial amplification, primer pairs corresponding to conserved sequences within the polymorphic regions of each gene were included in separate reactions. The product generated in the initial amplification was used as a template in five separate nested PCR reactions. In the nested reaction, separate primer pairs targeted the respective allelic types of *msp*-*1*(K1, MAD20 and RO33) and *msp*-*2* (IC/3D7 and FC27), with an amplification mixture containing 250 nM of each primer, 2 mM of MgCl_2_ and 125 µM of each dNTPs and 0.4 units Taq DNA polymerase (MyTaq^TM^ DNA Polymerase, Bioline). The cycling conditions in the thermocycler (TECHNE, GENIUS), for initial *msp*-*1* and *msp*-*2* PCR were as follows: 5 min at 95 °C, followed by 25 cycles for 1 min at 94 °C, 2 min at 58 °C and 2 min at 72 °C and final extension of 5 min at 72 °C. For msp-1 and msp-2 nested PCR, conditions were as follows: 5 min at 95 °C, followed by 30 cycles for 1 min at 95 °C, 2 min at 61 °C and 2 min at 72 °C and final extension of 5 min at 72 °C [49]. The allelic specific positive control 3D7 and DNA free negative controls were included in each set of reactions [48]. Fragment analysis of *msp*-*1* and *msp*-*2* amplified products were then performed through electrophoresis on 2 and 3% ethidium bromide-stained agarose gel, respectively, and after migration, the DNA fragments were visualized by UV trans- illumination. A standard curve is then drawn by measuring the distances traveled (in cm) from the well, of the bands of the size marker, according to the mathematical function: f (x) = y; where f (x) = the actual distance traveled by the band on the gel; y = log10 (bp). The size of an unknown strip is then determined by plotting the distance travelled on the x-axis, then projection on the coordinate axis to determine the size in base pairs. For individual samples, alleles were identified according to band size (Additional file 3: Figure S3; Additional file 4: Figure S4). This study assessed the frequency of the occurrence of each allele in the population. The study categorized clones into molecular weight groups differing by 20 bp for clear discrimination from other clones and elimination of errors that would result from estimating the molecular weight on agarose-gels.

### Data analysis

The *msp*-*1* and *msp*-*2* allele frequencies were expressed as the proportion of samples containing an allelic family compared to the total number of samples that gene was detected in isolates. The detection of one *msp*-*1* and *msp*-*2* allele was considered as one parasite genotype. The multiplicity of infection (MOI) was defined as the minimum number of *P. falciparum* genotypes per infected subject and estimated by dividing the number of amplified PCR fragments reflecting the parasite genotypes by the number of positive samples in the same marker [50]. The size of polymorphism in each allelic family was analysed; assuming that one band represented one amplified PCR fragment derived from a single copy of *P. falciparum msp*-*1*or *msp*-*2* genes. Alleles in each family were considered the same if fragment sizes were within 20 bp interval [16].

Spearman’s rank correlation coefficients were calculated to assess association between multiplicity of infection (MOI) and geometric mean parasite density and age. The heterozygosity index (He), which represents the probability of being infected by two parasites with different alleles at a given locus, was calculated by using the Genetic Analysis in Excel toolkit (GenAIEx) [51]. Briefly, the allelic diversity (*He*) for each antigenic markers was calculated based on the allele frequencies, using the formula:

$$He\, = \,\left[ {n/\left( {n - 1} \right)} \right]\,\left[ {\left( {1 - \sum pi^{2} } \right)} \right],$$

where n is the number of isolates sampled and pi is the allele frequency at a given locus. Allelic diversity has a potential range from 0 (no allele diversity) to 1 (all sampled alleles are different) [52]. Student’s test was used to compare MOI. The Chi square test or Fisher’s exact test was used for proportion comparisons. The *p* value < 0.05 was chosen as threshold significance for the various statistical tests. All statistical analyses were performed using SPSS version 20.0 (SPSS Inc., Chicago, IL, USA).

## Results

### Demographic and parasitological data

Of the 80 patients enrolled 57 (71.2%) were males, mean (± SD) age of participants was 20.96 (± 13.6) years. Participants had asexual parasitaemia ranging from 3699 to 14, 744 parasites/µL with a geometric mean of 12,513 parasites/µl (95% CI 12,167–12,859). The parasite DNA from the 80 *P. falciparum* samples was analysed for *msp*-*1* and *msp*-*2* genes. The estimated frequency of *msp*-*1* and *msp*-*2* gene amplification reactions with family-specific primers was 90% (72/80) and 86.3% (69/80), respectively.

### Allelic diversity of *P. falciparum msp*-*1* and *msp*-*2* genes

Polymorphism analysis was assessed in 80 *P. falciparum* isolates within the allelic families of *msp*-*1* and *msp*-*2* with a total of 253 distinct fragments detected. The *msp*-*1* gene analysis showed 63, 50, 31 fragments belonged to K1 (43.75% of overall detected *msp*-*1* alleles), MAD20 (34.72%) and RO33 (21.5%) allelic families, respectively. The *msp*-*2* gene analysis showed 58, 51 fragments belonged to FC27 (53.2% of overall detected *msp*-*2* alleles) and IC*/*3D7 (46.8%) allelic families, respectively.

The proportion of K1, MAD20 and RO33 types were 20.8, 4.2, and 4.2%, respectively. The remaining 70.8% (51/72) were polyclonal infections. Among polyclonal infections carrying two allelic types, the frequency of samples with K1/MAD20, K1/RO33, and MAD20/RO33 was 31.9, 5.6, and 5.6%, respectively. Infections with all three allelic types were detected in 29.2% of cases (Table 1).

**Table 1:** Genotyping of *P. falciparum msp*-*1* polymorphic region block 2 in malaria patients from Chewaka district, Ethiopia

*Msp*-*1*, N = 72	Frequency (%)	Allele size (bp)	No of alleles	Overall MOI
K1	15 (20.8)	130–300	6	2.0
MAD20	3 (4.2)	180–220	3	
RO33	3 (4.2)	150	1	
K1 + MAD20	23 (31.9)			
K1 + RO33	4 (5.6)			
MAD20 + RO33	3 (4.2)			
K1 + MAD20 + RO33	21 (29.2)			

*MOI* multiplicity of infection

Allele genotyping demonstrated the highly polymorphic nature (i.e. more alleles) of *P. falciparum* in Chewaka isolates with respect to *msp*-*1* and *msp*-*2* (Additional file 3: Figure S3 and Additional file 4: Figure S4). A total of 29 individuals with *msp* alleles were identified (10 for *msp*-*1* and 19 for *msp*-*2*). Among *msp*-*1* isolates, six K1 (130–300 bp), three MAD20 (180–220 bp) and one RO33 (150 bp) allelic families were noted.

In *msp*-*2*, a total of 19 different alleles were identified (Table 2), of which ten alleles belonged to FC27 and nine alleles belonged to IC/3D7. Allele sizes ranged from (260 to 540 bp) for FC27 and (170 to 450 bp) for IC/3D7 allelic families. The frequency of samples with only FC27 and IC/3D7 were 26.1% (18/69) and 15.9% (11/69), respectively. Forty of the isolates (58%) carried both *msp*-*2* allelic families.

**Table 2:** Genotype of *P. falciparum msp*-2 polymorphic region block region block 3 in malaria patients from Chewaka district, Ethiopia

*Msp*-*2*, N = 69	Frequency (%)	Allele size(bp)	No of alleles	Overall MOI
FC27	18 (26.1)	260–540	10	1.6
IC/3D7	11 (15.9)	170–450	9	
FC27 + IC/3D7	40 (58.0)			

*MOI* multiplicity of infection

### Genotype multiplicity of *P. falciparum* infection

Of the 80 positive samples, 64 (80%) harboured more than one parasite genotype identified by the presence of two or more alleles of one or both genes. with the overall mean MOI i.e., parasite clones per sample was 3.2 (95% CI 2.87–3.46). When considering *msp*-*1* and *msp*-*2* genes separately, the MOI was 2.0 (95% CI 1.82–2.18) and 1.6 (95% CI 1.46–1.70), respectively, while 51/72(70.9%) and 40/66(58%) of isolates contained multi-clonal infection at least with 2 clones, respectively. The heterozygosity index, which represents the probability of being infected by two parasites with different alleles at a given locus, was 0.43 for *msp*-*1*and 0.85 for *msp*-*2* loci. No significant correlation between multiplicity of infection and parasite density of patients (Spearman rank correlation = 0.094; p = 0.409) or multiplicity of infection and age (Spearman rank correlation = 0.072; p = 0.528). According to age, and parasite density the MOI was similar between individuals of different age and parasite density with-out significant difference (Table 3).

**Table 3:** MOI according age and parasite density in malaria patients from Chewaka district, Ethiopia

	MOI		
	*Msp1*	*Msp2*	*Msp*-*1 *+ *msp*-*2*
Age			
< 5 years	1.75	1.67	3.0
5–15 years	2.00	1.61	3.19
≥ 15 years	2.02	1.56	3.16
* P Value*	*p *>* 0.05*	*p *>* 0.05*	*p *>* 0.05*
Parasite density			
< 1000	2.50	1.25	3.75
≥ 10,000	1.97	1.60	3.13
* P Value*	*p *>* 0.05*	*p *>* 0.05*	*p *>* 0.05*

## Discussion

The genetic diversity of *P. falciparum* parasites impacts malaria transmission and malaria control strategies [53]. Genetic structures and population genetics studies of *P. falciparum* may hold the key for effective disease surveillance and control programmes, especially in Southwest Ethiopia as so far there is very limited information available on the genetic structures of *P. falciparum*. As the country moves towards malaria elimination, understanding the genetic diversity and population structure of the malaria parasite populations in hotspots is crucial to guide monitoring and evaluation of malaria control strategies and anti-malarial interventions. The present study provides a detailed assessment of genetic diversity and multiplicity of infection of *P. falciparum* parasites from Chewaka district, Southwest Ethiopia.

In this study, allele-specific PCR typing of *the msp*-*1* and *msp*-*2* loci showed considerably diverse and extensive allelic polymorphisms in *P. falciparum* populations in the analysed samples. However, the number of alleles may have been underestimated due to the limitations of the technique used. Indeed, the numbers of alleles (bands) detected may be underestimated due to sensitivity of the PCR technique used as minor fragments (< 50 bp) cannot be detected on the agarose gel and also similar sized fragments may be classified as identical leading to a false impression of similarity. Within allele families, alleles of the same size may have different amino acids motifs [51, 52], which emphasizes the importance of sequencing in future studies to confirm diversity and extensive allelic polymorphisms in the *P. falciparum*. A total of 10 and 15 different alleles for *msp*-*1* and *msp*-*2*, respectively, were obtained from the parasite isolates in Chewaka district, Ethiopia. This genetic diversity was consistent with the diversity found in Kolla-Shele area, Southwest Ethiopia (*msp*-*1*: 11; *msp*-*2*: 12) in 2015 [42], in Northwest Ethiopia (*msp*-*1*: 12; *msp*-*2*: 22) in 2018 [43], and Brazzaville in the Republic of Congo (*msp*-*1*: 15; *msp*-*2*: 20) in 2018 [54]. In contrast, a higher diversity (*msp*-*1*: 26; *msp*-*2*: 25) was found in Bioko Island, Equatorial Guinea in 2018, even though this area has comparable malaria endemicity patterns [53]. K1 was the predominant allelic family for *msp*-*1* as also demonstrated in previous studies in Africa, including Southwest Ethiopia [42], Brazzaville, Republic of Congo [16] and Gabon [55]. However, in studies conducted in Northern Ethiopia [44], Central Sudan [14] and Bioko Island, Equatorial Guinea [53] the MAD-20 allele was found to be predominant.

In this study, the RO33 family showed no polymorphism with only a single allele (160 bp). This is similar to findings in Congo [16]. Allele typing of *msp*-*2* showed that FC27 was the predominant allelic family as also demonstrated in previous reports from Benin [56] and Central Sudan [14], but in contrast with previous studies in Ethiopia [42] and Brazzaville, Republic of Congo [16]. A variation in the prevalence of alleles between different studies likely reflects the differences in sample population. Thus, it is important to conduct studies that include adequate sample size as well as sampling at different time point within the same region to assess and compare the genetic profile of parasites circulating in endemic areas in an attempt to avoid intra and inter individual variation in the number of parasite genotypes detected in the different episodes of malaria. Besides, methodological differences may also affect the comparability of results. Hence, further investigations with more powerful techniques such as capillary electrophoresis and DNA sequencing are needed to better characterize the malaria parasites in the country.

Multiplicity of infection (MOI), i.e. the number of different *P. falciparum* strains co-infecting a single host, has been shown to be a common feature in most malaria-endemic areas and was reported to vary with age, parasite density, immune status, epidemiological settings and transmission intensity [57–60]. In this study, 80% of the isolates harboured more than one parasite genotype identified by the presence of two or more alleles of one or both genes with the overall mean MOI being 3.2 (95% CI 2.87–3.46). The overall MOI value reported in this study was higher than previously reported studies, including Ethiopia (MOI: 1.8–2.6) between 2015 and 2018 [42–44], Brazzaville, Republic of Congo (MOI: 2.2) [16] in 2011 and Bobo-Dioulasso, Burkina Faso (MOI: 1.95) [61]. In contrast to study reported in Bioko Island, Equatorial Guinea (MOI: 5.51) [53] in 2018 and Gabon (MOI: 4.0) [62] in 2018. The difference in MOI can be explained by the differences in intensity of malaria transmission seasons. In this study, samples were collected during the major malaria transmission season of September to December, when malaria transmission is very intense. All year round (seasonal) studies covering major and minor transmission seasons are needed to better understand genetic profiles in this area including a sense on seasonal variations.

The results of this study show that age has no association on multiplicity of infection similar to other studies [42, 44, 50], but in contrast with reports from Brazzaville, Republic of Congo [53] and Central Sudan [14]. Previous studies regarding the variation of MOI over age have suggested that the influence of age on the multiplicity of infection is highly affected by endemicity of malaria [56–59]. This is probably a reflection of the development of anti-parasite specific immunity [31]. Thus, in holo- or hyperendemic areas, immunity develops faster and at younger age than in areas with less intense transmission [63]. Studies have shown an age-dependent MOI in a village with intense perennial malaria transmission but not in areas where malaria is mesoendemic [50, 58]. Similarly, in this study reported that no significant relation between MOI and the parasite count, similar to reports from previous studies in Ethiopia [42, 44], but in contrast with reports from Bioko Island, Equatorial Guinea [56]. This may have been due to the small number of isolates analysed.

High transmission regions like those in many African countries are commonly characterized by *P. falciparum* populations that are genetically diverse. Antigenic marker genotyping carried out in African regions like Burkina Faso, Sao Tome, Malawi, Uganda and Tanzania have identified *P. falciparum* populations with alleles occurring at a frequency below 10 percent with a very high *He* level of 0.78 to 0.99 [17]. This study indicate that the genetic diversity values were higher based on heterozygosity index for *msp*-*2* (He = 0.85), than for *msp*-*1* (He = 0.43), suggesting a large genotype diversity within the *msp*-*2* locus, which was higher than previously reported from Northwest Ethiopia (*msp*-*2*: *He* 0.62) in 2018 [44]. Djibouti, a neighbouring country to Ethiopia, an initially moderate level of genetic diversity declined over an 11-year period to the point that the expected heterozygosity reached zero in 2009 consistent with very low diversity [30].

Despite the lack of entomological data from Chewaka district, the number of clones co-infecting a single host can be used as an indicator of the level of malaria transmission or the level of host acquired immunity [57, 64, 65]. Besides, transmission intensity can also be affected by other factors, such as vector biting behaviour and endemicity [64]. Inferring high transmission intensity from the presence of multi-clonal infections alone has additional limitations including estimates of MOI varying by genotyping method, potential impact from sampling frequency and a non-linear relationship between MOI and transmission intensity [64]. Despite these limitations, infections with multiple clones observed in this study, combined with evidence of high genetic diversity may indicate high transmission intensity in the study area.

## Limitations of the study

The limitation of the present study is the small number of isolates analysed, which were collected during a therapeutic efficacy study of artemether-lumefantrine (Coartem^®^) in the region. In this study, the association between the dominant allelic families and the manifestation of the disease was not examined because all samples were collected from uncomplicated malaria patients. Thus, the relationship between malaria severity or clinical symptom and genetic diversity could not be addressed in the present study. The collection of samples throughout the year (not just in high transmission season) would potentially give a better understanding of the true diversity in the region. Despite these limitations, the data from the present study has confirmed the high genetic diversity profile and mixed-strain infections of *P. falciparum* populations in Chewaka district, Ethiopia, potentially reflecting both the endemicity level as well as the fact that malaria transmission remains high in Southwest, Ethiopia.

## Conclusions

The high level of polyclonal infections with *P. falciparum* parasites harbouring multiple genotypes and also infections with high MOI study indicate the extensive genetic diversity and complexity of *P. falciparum* infection in the region. More effort is needed to control malaria transmission and prevent the emergence of resistance alleles in the study area.

## Supplementary information

**Additional file 1: Table S1.** Primer sequence used for PCRs to screen and genotype samples collected in study of genotyping diversity of *P. falciparum* parasites in Chewaka district, Ethiopia.

**Additional file 2: Table S2.** Primer sequence used for PCRs to screen and genotype samples collected in study of genotyping diversity of *P. falciparum* parasites in Chewaka district, Ethiopia.

**Additional file 3: Figure S3.** Prevalence of *Plasmodium falciparum* msp-1 alleles in clinical isolates from Chewaka district, Ethiopia.

**Additional file 4: Figure S4.** Prevalence of *Plasmodium falciparum* msp-2 alleles in clinical isolates from Chewaka district, Ethiopia.

## Data Availability

The data sets generated and/or analysed during the current study are included within the article and its additional files.
