# Supplementary material for: Genetic diversity and genotype multiplicity of Plasmodium falciparum infection in patients with uncomplicated malaria in Chewaka district, Ethiopia
Source: Malar J. 2020 Jun 8;19:203. doi: 10.1186/s12936-020-03278-6 (PMC7281928; doi:10.1186/s12936-020-03278-6)
Supplement: Supplementary file 1 — Additional file 1: Table S1. Primer sequence used for PCRs to screen and genotype samples collected in study of genotyping diversity of P. falciparum parasites in Chewaka district, Ethiopia. [file 12936_2020_3278_MOESM1_ESM.docx]

Table S1: Primer sequence used for PCRs to screen and genotype samples collected in study of genotyping diversity *of P. falciparum* parasites in Chewaka district, Ethiopia

| PCR | Locus | Allele | Primer | | Sequence  (primer differences are shown in bold, underlined font) | Reference |
| --- | --- | --- | --- | --- | --- | --- |
|  |  |  | Forward | Reverse |  |  |
| pPCR | *msp1* | N/A | ✓ |  | CTAGAAGCTTTAGAAGATGCAGTATTG | ^[[1]](#footnote-1)^ |
|  |  |  |  | ✓ | **C**TTAAATAGTATTCTAATTCAAGTGGATCA |  |
| nPCR |  | K1 | ✓ |  | **A**AATGAAGAAGAAATTACTACAAAAGGTGC |  |
|  |  |  |  | ✓ | **G**CTTGCATCAGCTGGAGGGCTTGCACCAGA |  |
|  |  | MAD20 | ✓ |  | AAATGAAGGAACAAGTGGAACAGCTGTTAC |  |
|  |  |  |  | ✓ | ATCTGAAGGATTTGTACGTCTTGAATTACC |  |
|  |  | RO33 | ✓ |  | TAAAGGATGGAGCAAATACTCAAGTTGTTG |  |
|  |  |  |  | ✓ | **CAAGTAATTTTGAACTCTATGTTTTAAATCAGCGTA**  CATCTGAAGGATTTGCAGCACCTGGAGATC |  |

1. WHO primers are taken from (World Health Organization, 2007). [↑](#footnote-ref-1)
