# Supplementary material for: Genetic diversity and genotype multiplicity of Plasmodium falciparum infection in patients with uncomplicated malaria in Chewaka district, Ethiopia
Source: Malar J. 2020 Jun 8;19:203. doi: 10.1186/s12936-020-03278-6 (PMC7281928; doi:10.1186/s12936-020-03278-6)
Supplement: Supplementary file 2 — Additional file 2: Table S2. Primer sequence used for PCRs to screen and genotype samples collected in study of genotyping diversity of P. falciparum parasites in Chewaka district, Ethiopia. [file 12936_2020_3278_MOESM2_ESM.docx]

Table S2: Primer sequence used for PCRs to screen and genotype samples collected in study of genotyping diversity *of P. falciparum* parasites in Chewaka district, Ethiopia

| PCR | Locus | Allele | Primer | | Sequence  (primer differences are shown in bold, underlined font) | Reference |
| --- | --- | --- | --- | --- | --- | --- |
|  |  |  | Forward | Reverse |  |  |
| pPCR | *msp2* | N/A | ✓ |  | ATGAAGGTAATTAAAACATTGTCTATTATA | ^[[1]](#footnote-1)^ |
|  |  |  |  | ✓ | CTTTGTTACCATCGGTACATTCTT  *ATATGGCAAAAGATAAAACAAGTGTTGCTG* |  |
| nPCR |  | FC27 | ✓ |  | **GCTTATAATATGAGTATAAGGAGAA**  AATACTAAGAGTGTAGGTGCARATGCTCCA  *GCAAATGAAGGTTCTAATACTAATAG* | ^[[2]](#footnote-2)^ |
|  |  |  |  | ✓ | TTTTATTTGGT**GCATTGCCAGAACTTGAA**C  *GCTTTGGGTCCTTCTTCAGTTGATTC* |  |
|  |  | 3D7/IC | ✓ |  | **GCTTATAATATGAGTATAAGGAGAA**  AGAAGTATGGCAGAAAGTAAKCCTYCTACT  *GCAGAAAGTAAGCCTTCTACTGGTGCT* |  |
|  |  |  |  | ✓ | **CTGAAGAGGTACTGGTAG**  GATTGTAATTCGGGGGATTCAGTTTGTTCG  *GATTTGTTTCGGCATTATTATGA* |  |

1. World Health Organization, 2007 [↑](#footnote-ref-1)
2. Falk, Maire, Sama, Owusu-Agyei, Smith, & Beck, 2006) and (Zwetyenga, Rogier, Tall, Fontenille, Snounou, & Trape, 1998) [↑](#footnote-ref-2)
