# Supplementary material for: Genetic diversity and genotype multiplicity of Plasmodium falciparum infection in patients with uncomplicated malaria in Chewaka district, Ethiopia
Source: Malar J. 2020 Jun 8;19:203. doi: 10.1186/s12936-020-03278-6 (PMC7281928; doi:10.1186/s12936-020-03278-6)
Supplement: Supplementary file 3 — Additional file 3: Figure S3. Prevalence of Plasmodium falciparum msp-1 alleles in clinical isolates from Chewaka district, Ethiopia. [file 12936_2020_3278_MOESM3_ESM.docx]

Figure S3 Prevalence of *Plasmodium falciparum* *msp-1* alleles in clinical isolates from Chewaka district, Ethiopia
